# Plasma Rivaroxaban Level in Patients With Early Stages of Chronic Kidney Disease—Relationships With Renal Function and Clinical Events

**DOI:** 10.3389/fphar.2022.888660

**Published:** 2022-05-17

**Authors:** Chun-Fung Sin, Ka-Ping Wong, Hoi-Man Wong, Chung-Wah Siu, Desmond Y. H. Yap

**Affiliations:** ^1^ Department of Pathology, Queen Mary Hospital, The University of Hong Kong, Hong Kong, Hong Kong SAR, China; ^2^ Division of Cardiology, Department of Medicine, Queen Mary Hospital, The University of Hong Kong, Hong Kong, Hong Kong SAR, China; ^3^ Division of Nephrology, Department of Medicine, Queen Mary Hospital, The University of Hong Kong, Hong Kong, Hong Kong SAR, China

**Keywords:** rivaroxaban, plasma level, renal function, bleeding, chronic kidney disease

## Abstract

Introduction Drug accumulation of rivaroxaban is a concern in patients with chronic kidney disease (CKD). Data regarding the plasma rivaroxaban levels in early CKD patients and its relationship with clinical events is lacking. Methods Early CKD patients (Stage 1–3) with atrial fibrillation who received rivaroxaban (15 or 20 mg daily) were recruited. Plasma rivaroxaban levels were measured at 2 hours (peak) and 24 hours (trough) after drug administration, and correlated with eGFR and clinically significant events during the follow-up period (1 January 2018 to 31 October 2021). Results Ninety-two patients were included (CKD stage 1 *n*=10, stage 2 *n*=53, stage 3 *n*=29). Plasma trough levels in patients with stage 3 CKD were significantly higher than those with stage 2 and 1 CKD (66.0±34.9 ng/ml vs. 35.7 ± 24.7 ng/ml vs. 34.7 ± 26.2 ng/ml, respectively, *p*=0.005), and showed inverse relationship with eGFR (r=0.391, p=0.001) in patients receiving 20 mg daily. The plasma trough rivaroxaban level correlated with PT and APTT (r = 0.650 and 0.44, respectively, *p*<0.001 for both). Plasma trough rivaroxaban level in those with bleeding were higher than those who did not (59.9 ± 35.6 ng/ml vs. 41.1 ± 29.2 ng/ml, *p*=0.011), and multivariate analysis suggested that plasma trough rivaroxaban level was associated with the rate of bleeding complications (OR: 1.020, 95% CI 1.002-1.038, p=0.028). Conclusion Plasma trough rivaroxaban levels correlated with renal function in early CKD patients, and its measurement may help dosage optimization in patients with renal impairment. Moreover, our data suggests that there may be an association between plasma trough rivaroxaban level and the rate of bleeding complication

## Introduction

Atrial fibrillation (AF) is an important risk factor for ischaemic stroke and transient ischaemic attack. Anticoagulants are essential for the prophylaxis of stroke in patients with AF and direct oral anticoagulants (DOACs) are increasingly popular in this clinical context. Rivaroxaban is a direct Xa inhibitor that exhibit comparable clinical efficacy for stroke prevention as warfarin (Patel et al., 2011; Blumer et al., 2021), and results from the ROCKET-AF trial showed that rivaroxaban was associated with lower risk of fatal bleeding compared with warfarin (Patel et al., 2011).

Renal excretion constitutes 36% of drug elimination of rivaroxaban, and hence its use in patients with chronic kidney disease (CKD) can be challenging and previous clinical trials of rivaroxaban have also excluded patients with creatinine clearance (CrCl) < 30 ml/min (Chan et al., 2016). Although pharmacokinetic studies have demonstrated rivaroxaban accumulation in patients with renal impairment who received a dose of 10 mg daily (Kubitza et al., 2010), previous studies have shown conflicting results regarding the risk of bleeding in patients with renal impairment (Mao et al., 2014; Weir et al., 2020; Wetmore et al., 2020; Ashton et al., 2021; Atarashi et al., 2021). Chromogenic assay of anti-Xa activity is one of the methods for measuring plasma rivaroxaban level, (Conway et al., 2017), and has emerged as a useful means to optimize the dosage and minimize side effects. Previous studies have reported that the peak and trough rivaroxaban levels can vary considerably among individuals receiving rivaroxaban (Douxfils et al., 2018; Lin et al., 2019). While rivaroxaban accumulation is an important concern in patients with renal impairment, there is limited data regarding the relationship between plasma rivaroxaban level and renal function in a real-world situation. It is also recognized that the thrombotic and bleeding tendency varies with ethnicities, for instance Asians showed higher anticoagulant-related intracranial haemorrhage compared with Caucasians (Shen et al., 2007; Wong et al., 2014). In this context, the relationship between the plasma rivaroxaban level and bleeding complications in Chinese patients with early stages of chronic kidney disease (CKD) have not been studied. The primary objective of this study was to investigate the relationships between plasma rivaroxaban levels and renal dysfunction in patients with early stages of CKD. Other exploratory outcomes include the potential relationship between plasma rivaroxaban levels and the rates of thromboembolic and haemorrhagic events in Chinese patients with early CKD.

## Methods

### Patients and Retrieval of Clinical Data

This prospective study was approved by Institutional Review Board of the University of Hong Kong/Hospital Authority Hong Kong West Cluster (IRB HKU/HAHKWC) (Reference number: UW 22-086). Patients with history AF receiving a stable dose of rivaroxaban were recruited from the outpatient clinics at the Department of Medicine, Queen Mary Hospital of Hong Kong. All patients provided written consent. Rivaroxaban was prescribed at dosages of 20 mg daily or 15 mg daily, according to the renal function or assessment of patients’ medical conditions by the attending physician. In general, patients with CrCl≤50 ml/min (as determined by the CKD-EPI equation) at the time of stating rivaroxaban were prescribed a reduced dose of 15 mg daily. Patients with non-Chinese ethnic origin and poor drug compliance (defined by less than 90% of compliance) were excluded from the study. At study enrollment, data on patient demographics, medical comorbidities, liver and renal biochemistry, clotting profiles were collected. The drug compliance to rivaroxaban treatment by pill-counting during outpatient clinic follow up was documented in the medical records. Patients were followed at the medical outpatient clinics every 8–12 weeks, depending on clinical situation. Dosages of rivaroxaban, concomitant medications, renal function (serum creatinine, estimated GFR by MDRD equation) and clinically significant events (i.e., thromboembolic events or bleeding complications) were documented in medical record during each follow-up. Severity of chronic kidney disease was classified according to Kidney Disease: Improving Global Outcomes (KDIGO) classification (Levey et al., 2005). As the clinicians have chosen the dosage according to the eGFR values provided by our hospital laboratory (calculated by MDRD), we have also analysed our data using CrCl estimated by the Cockroft-Gault (CG) equation to ensure consistency of our results. ISTH definition of major and minor bleeding was used. Briefly, major bleeding was defined as Hb level below 8 g/dl or drop of Hb more than 2 g/dl from baseline, limb threatening or life-threatening bleeding. All other bleeding episodes were defined as minor bleeding (Kaatz et al., 2015). Thromboembolic events were defined as clinical evidence of transient ischaemic attached (TIA) or stroke, as well as radiological evidence of new onset cerebrovascular accident (CVA) or venous thromboembolism. Clinically significant bleeding and thromboembolic complications were adjudicated independently by investigators who were unaware of the levels of rivaroxaban. Clinical data were first retrieved from the electronic health systems (ePR of the Hong Kong Hospital Authority) and verified by review of patient case records. The follow-up period of recurrent ischaemic/thromboembolic events and bleeding complications was 1 January 2018 to 31 October 2021.

### Blood Sampling and Processing

To ensure a stable plasma rivaroxaban concentration, blood samples were measured in patients after receiving rivaroxaban for at least a week (more than five half-life). The peak level was collected after 2 h of rivaroxaban administration and the trough level was taken 24 h after last dose of rivaroxaban. Blood samples were taken by vacuum plastic tubes containing 3.2% trisodium citrate, followed by centrifugation at 3,750 rpm for 10 min. Platelet poor plasma were separated and stored at or below −70°C for future use.

### Measurement of Plasma Rivaroxaban Level and Coagulation Parameters

Plasma rivaroxaban level was measured by BIOPHEN DiXal kit (Hyphen BioMed, France) in Sysmex CS5100 analyzer according to the manufacturer’s instructions. The assay utilized chromogenic anti-Xa assay specifically calibrated for rivaroxaban and plasma rivaroxaban level was expressed as unit ng/ml. Rivaroxaban level measurement was performed after the value obtained by quality control materials fell within specific ranges provided by manufacturers. Coagulation screening tests namely [prothrombin time (PT) and activated partial thromboplastin time (APTT)] were performed in Sysmex CS5100 analyzer. The reagents used for PT and APTT measurements were Thromorel S Reagent and Actin FSL Activated PTT Reagent respectively (both manufactured by Siemens).

### Data Analysis and Statistical Analysis

Continuous variables were expressed as mean ± S.D. or median (range) as appropriate. The differences in continuous variables between groups were assessed by using one-way ANOVA (for parametric test) or independent-samples Kruskal–Wallis test (for non-parametric test). Categorical variables were expressed as frequencies and percentages, and they were analyzed by chi-square test or Fisher’s exact test where appropriate. Pearson correlation was used to determine the relationship between plasma rivaroxaban level with PT and APTT. Linear regression was used to examine the relationship between plasma rivaroxaban level and other clinical parameters. Logistic regression was used to determine the relationship between rate of bleeding complications, recurrent ischaemic/thromboembolic events and plasma rivaroxaban level as well as other clinical parameters including dosage, age, eGFR, presence of DM, hypertension, history of ischaemic stroke and ischaemic heart disease. Univariate regression analysis was performed first to evaluate the relationship between individual parameters, followed by multivariate analysis.

The primary outcome was defined as the relationship between plasma rivaroxaban level and renal function. The secondary outcome was the association between plasma rivaroxaban level and clinically significant events. In order to establish a correlation (r = -0.3) between plasma rivaroxaban level and eGFR, a sample size of 85 will achieve a 80% power at a 95% confidence interval (95% CI). Based on previous studies that reported a 21% event rate for all major and non-major bleeding (Wong et al., 2014), a sample size of 150 patients will achieve 80% power to determine the relationship between plasma rivaroxaban level and bleeding events by univariate logistic regression at a 95% CI.

All statistical analysis was performed by using IBM SPSS software version 27 and *p*-values of less than 0.05 were considered statistically significant.

## Results

### Patient Characteristics

A total of 92 Chinese patients (CKD stage 1 *n* = 10, stage 2 *n* = 53 and stage 3 *n* = 29) were recruited into the study (Table 1). The primary indication of rivaroxaban was AF. Sixty-eight patients (73.9%) received a dose of 20 mg daily and 24 patients (26.1%) received a dose of 15 mg daily. All recruited patients showed good drug compliance (i.e., > 90% compliance by pill-counting). The time taken from starting rivaroxaban to blood taking for plasma rivaroxaban level was at least 1 month apart, with a median time period of 13 months (range: 1–32 months). Patients receiving rivaroxaban 15 mg daily were older and showed higher CHADS_2_ score, lower eGFR and higher incidence of hypertension compared with those taking rivaroxaban 20 mg daily (*p* < 0.05 for all). Twenty-four patients received rivaroxaban 15 mg daily. Dosage in these patients was reduced because of patient’s clinical condition (18 related to impaired renal function and six because of mild bleeding symptoms at the time of rivaroxaban initiation). When CrCl were estimated by the CG equation, 22 out of 68 patients (32.4%) who received rivaroxaban 20 mg daily should in fact receive 15 mg daily instead. Diabetes nephropathy and hypertensive nephropathy were the commonest causes of renal impairment for our patients (79.3% in total).

**TABLE 1:** Baseline characteristics of patients receiving rivaroxaban 15 and 20 mg daily.

		Rivaroxaban 15 mg (*n* = 24)	Rivaroxaban 20 mg (*n* = 68)	*p*-value	Overall (*n* = 92)
Demographic information					
Sex (male) (%)		10 (41.7)	47 (69.1)		57 (62.0)
Age, years old, mean (range)		75.46 (60-86)	68.3 (39-95)	0.002	70.2 (39-95)
CHADS2 (mean±SD)		2.17 ± 1.13	1.29 ± 1.13	<0.001	1.52 ± 1.19
Stages of chronic kidney disease (%)	1	0 (0)	10 (14.7)	—	10 (11.0)
	2	9 (34.6)	44 (64.7)	—	53 (56.7)
	3	15 (57.7)	14 (20.6)	—	29 (31.6)
Laboratory parameters					
eGFR by MDRD equation (ml/min/1.73m2), mean (range)		54.33 (31-88)	74.03 (41 to >90)	<0.001	68.63 (31 to >90)
eGFR by Cockcroft-Gault formula (ml/min/1.73m2), mean (range)		45.10 (23-75)	66.17 (29 to >90)	<0.001	60.68 (23 to >90)
eGFR <50 ml/min/1.73m2 by MDRD equation (%)		11 (45.8)	4 (5.9)	0.001	15 (16.3)
eGFR <50 ml/min/1.73m2 by Cockcroft-Gault formula (%)		15 (62.5)	22 (32.4)	0.004	37 (40.2)
Creatinine (µmol/L), mean (range)		104.21 (62-186)	86.85 (51-141)	0.002	91.38 (51-186)
PT (s), mean (range)		12.8 (10.6-15.4)	12.3 (10.5-19.5)	0.113	12.4 (10.5-19.5)
APTT (s), mean (range)		31.5 (23.8-35.9)	31.5 (26.4-46.4)	0.987	31.4 (23.8-46.4)
Medical comorbidity (%)					
Diabetes mellitus (%)		11 (45.8)	19 (27.9)	0.089	30 (32.6)
Hypertension (%)		20 (83.3)	41 (60.3)	0.047	61 (66.3)
Hyperlipidemia (%)		9 (37.5)	13 (19.1)	0.095	22 (23.91)
Ischemic heart disease (%)		2 (8.33)	7 (13.2)	1.000	9 (9.78)
Old stroke/cerebrovascular accidents (%)		3 (12.5)	7 (10.3)	0.720	10 (10.9)
Past history of cancer (%)		4 (16.7)	8 (11.8)	0.726	12 (13.0)
Causes of chronic kidney impairment					
Diabetic nephropathy (%)		8 (53.3)	5 (35.7)	—	13 (44.8)
Hypertensive nephropathy (%)		6 (40.0)	4 (28.6)	—	10 (34.5)
Unknown cause (%)		1 (6.67)	5 (35.7)	—	6 (20.7)
Indications of rivaroxaban		—	—	—	—
Atrial fibrillation		24 (100)	68 (100)	—	92 (100)

### Relationship Between Plasma Rivaroxaban Level and Dosage

Despite receiving a lower dose of rivaroxaban, patients receiving 15 mg daily showed a significantly higher plasma trough level when compared with those patients taking 20 mg daily (60.4 ± 35.8 ng/ml vs. 41.8 ± 29.6 ng/ml, *p* = 0.014) (Table 2). The peak rivaroxaban level was also numerically higher in those receiving 15 mg daily compared with those taking 20 mg daily, although statistical significance was not reached (318.1 ± 127.1 ng/ml vs. 272.6 ± 119.9 ng/ml, *p* = 0.126).

**TABLE 2:** Peak and trough rivaroxaban level in patients receiving different dosage of rivaroxaban.

	Rivaroxaban 15 mg (*n* = 24)	Rivaroxaban 20 mg (*n* = 68)	*p*-value	Overall (*n* = 92)
Peak rivaroxaban level (ng/ml), mean ± SD	318.4 ± 127.1	272.6 ± 119.9	0.126	284.3 ± 122.7
Trough rivaroxaban level (ng/ml), mean ± SD	60.4 ± 35.8	41.8 ± 29.6	0.014	46.6 ± 32.18

### Association Between Plasma Rivaroxaban Level and Renal Function

**FIGURE 1:**
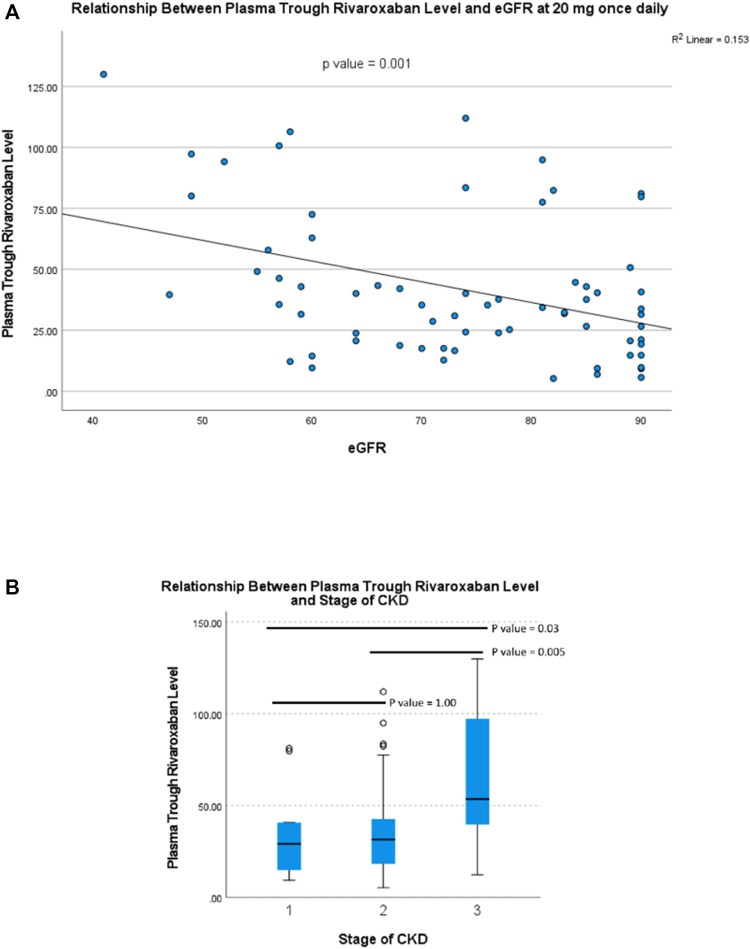
Relationship between plasma trough rivaroxaban level and **(A)** eGFR and **(B)** stage of CKD in patients receiving rivaroxaban 20 mg once daily.

**FIGURE 2:**
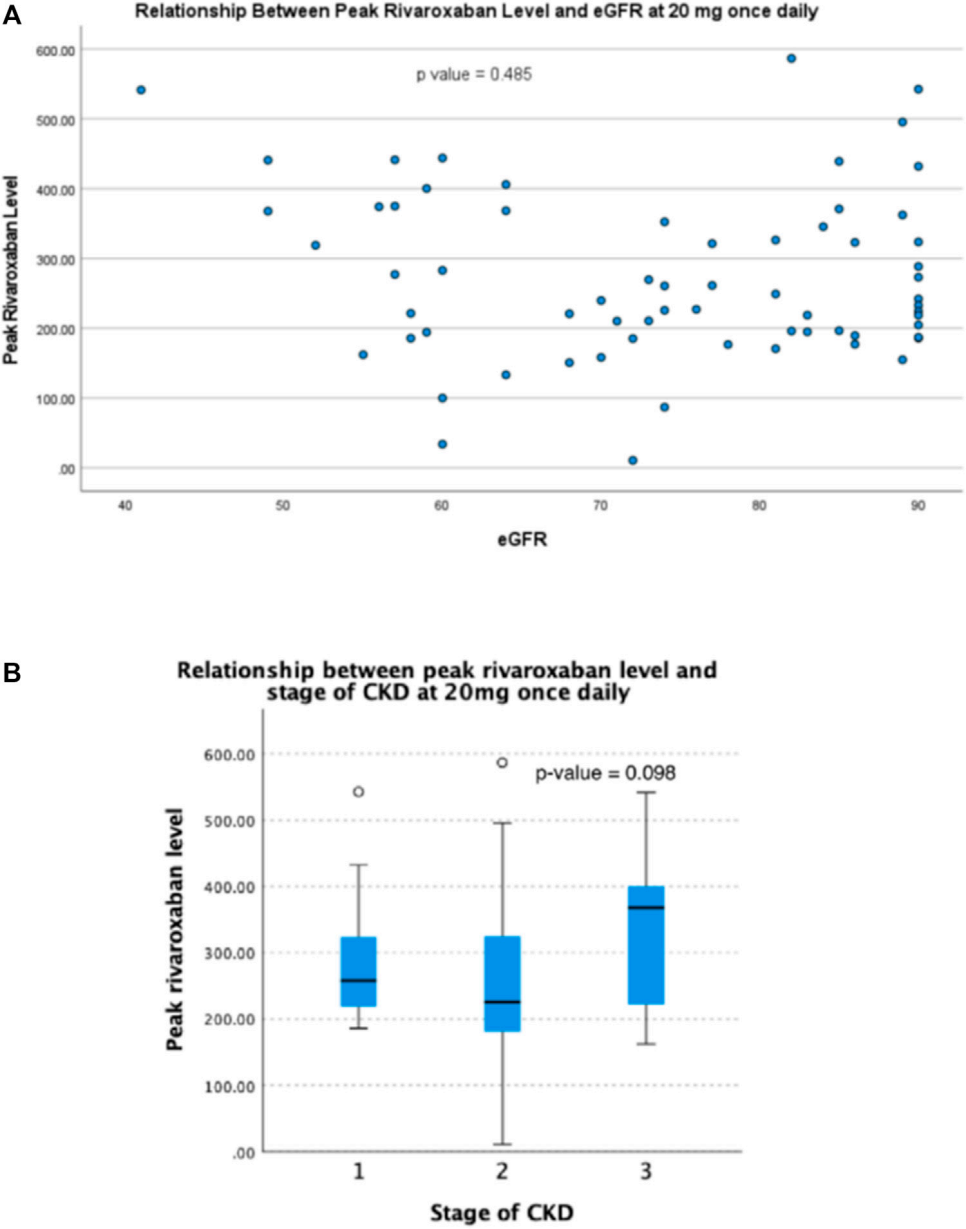
Relationship between peak plasma rivaroxaban level and **(A)** eGFR and **(B)** stage of CKD in patients receiving rivaroxaban 20 mg once daily.

For patients taking rivaroxaban 20 mg daily, the plasma trough level was inversely correlated with eGFR (r = 0.391, *p* = 0.001) (Figure 1A). Plasma trough levels in patients with stage 3 CKD were significantly higher than those with stage 2 and 1 CKD (66.0 ± 34.9 ng/ml vs. 35.7 ± 24.7 ng/ml vs. 34.7 ± 26.2 ng/ml, respectively, *p* = 0.005) (Figure 1B). However, there was no significant correlation between plasma peak level and eGFR (r = -0.087, *p* = 0.485) (Figure 2A). The plasma peak level was numerically higher in those patients with CKD stage 3 when compared with stage 2 and stage 1, although not reaching statistical significance (330.8 ± 116.1 ng/ml vs. 250.5 ± 118.1 ng/ml and 291.8 ± 114.7 ng/ml in patients with stage 3, 2 and 1 CKD respectively, *p* = 0.098) (Figure 2B).

**FIGURE 3:**
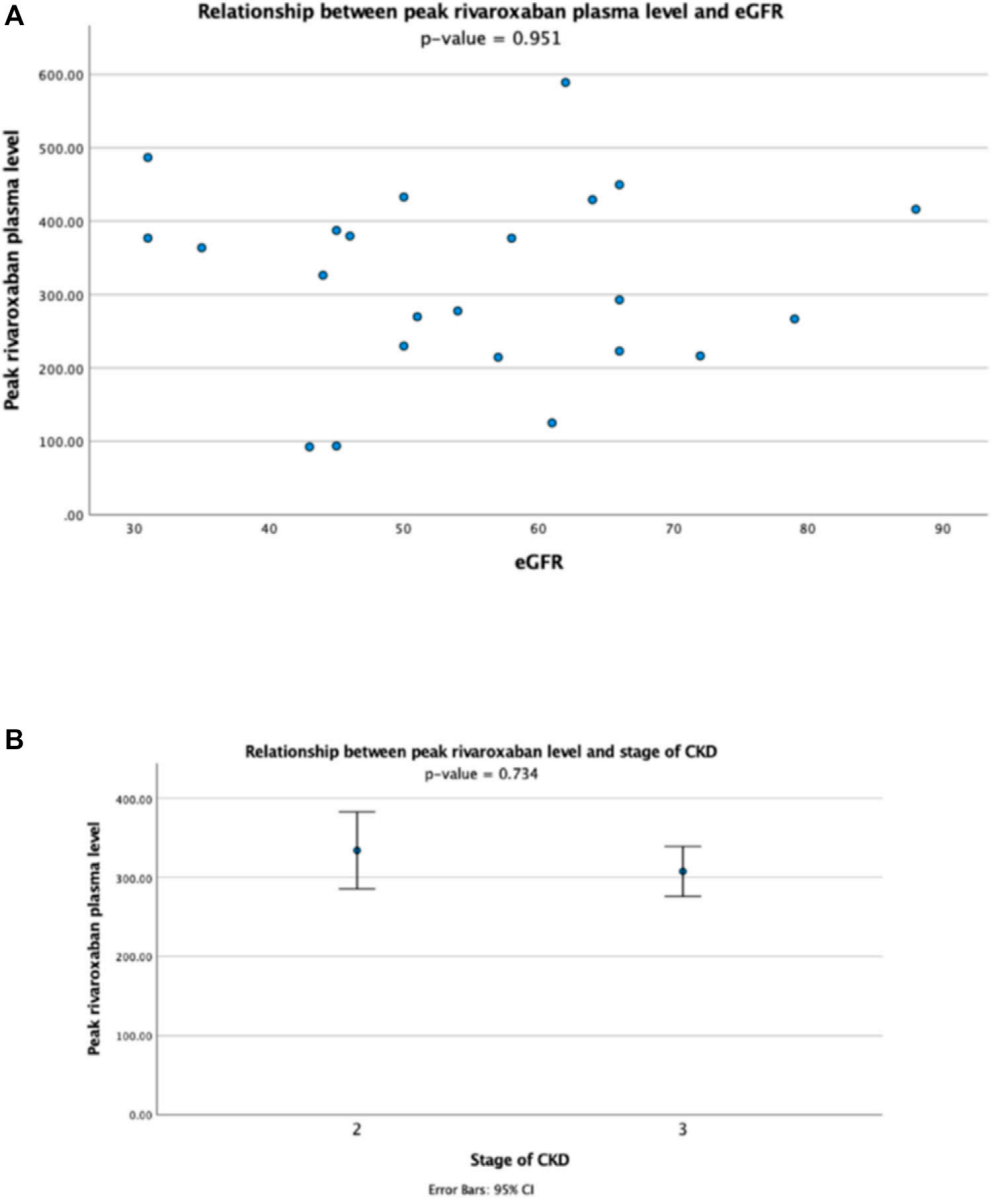
Relationship between peak plasma rivaroxaban level and **(A)** eGFR and **(B)** stage of CKD in patients receiving rivaroxaban 15 mg once daily.

**FIGURE 4:**
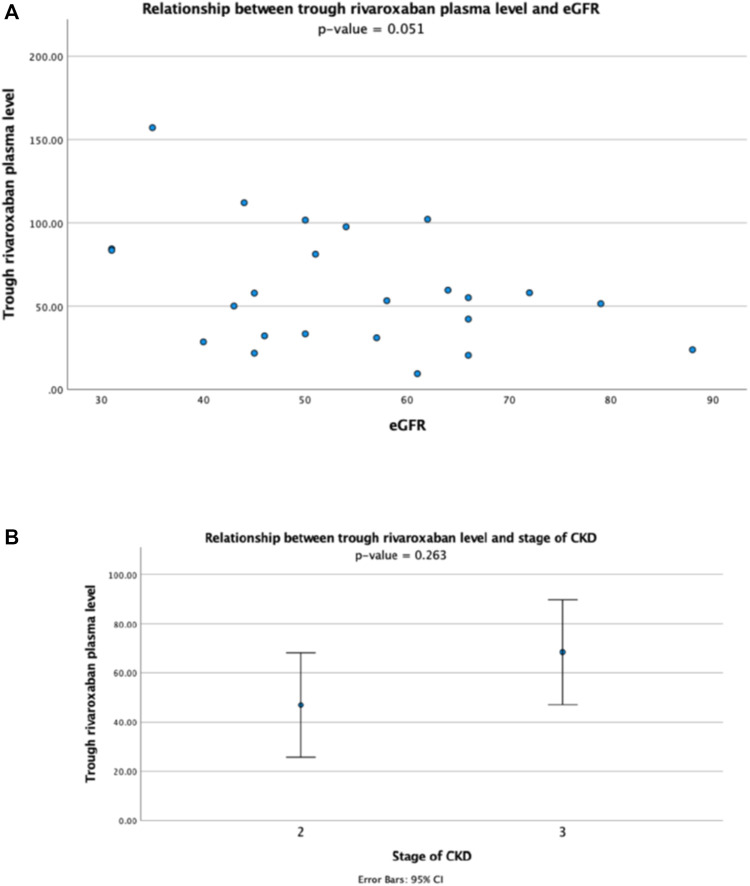
Relationship between trough plasma rivaroxaban level and **(A)** eGFR and **(B)** stage of CKD in patients receiving rivaroxaban 15 mg once daily.

For patients receiving rivaroxaban 15 mg daily, there was no correlation between the plasma peak level and eGFR (r = -0.014, *p* > 0.1) (Figure 3A), and the plasma peak levels also showed no difference between patients with different stages of CKD (Figure 3B). The plasma trough level showed a trend of inverse correlation with eGFR though statistically not significant (r = -0.402, *p* = 0.051) (Figure 4A), and the trough plasma rivaroxaban levels were also numerically higher in patients with CKD stage 3 when compared with stage 2 (68.4 ± 38.5 ng/ml vs. 47.0 ± 27.6 ng/ml, *p* > 0.05) (Figure 4B).

### Relationship Between Plasma Rivaroxaban Level and Clotting Parameters

**FIGURE 5:**
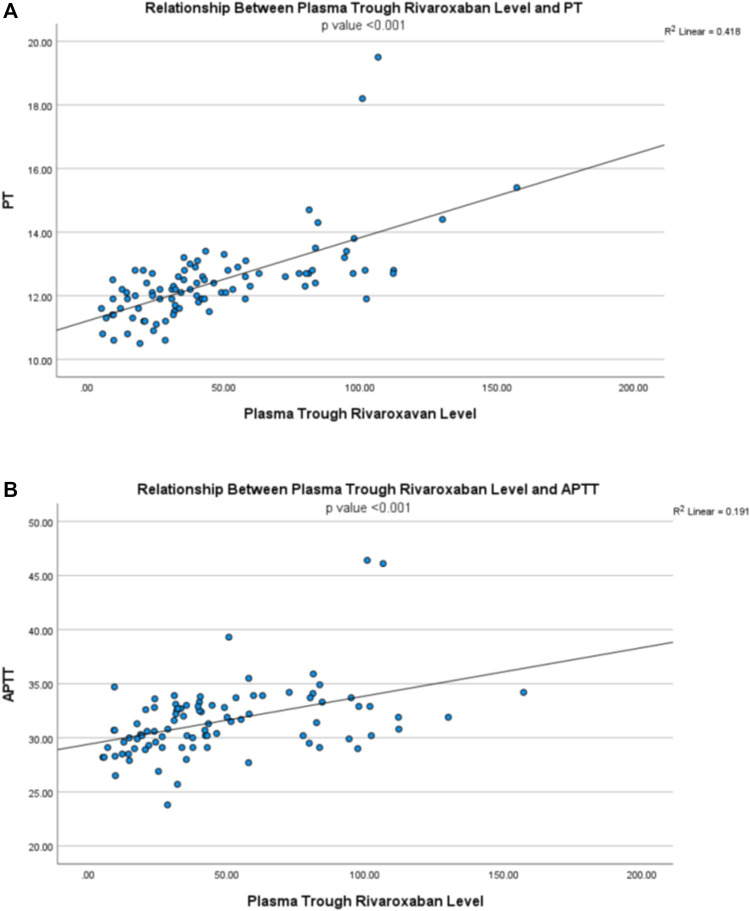
Relationship between plasma trough rivaroxaban level and **(A)** prothrombin time (PT) and **(B)** activated partial thromboplastin time (APTT).

**FIGURE 6:**
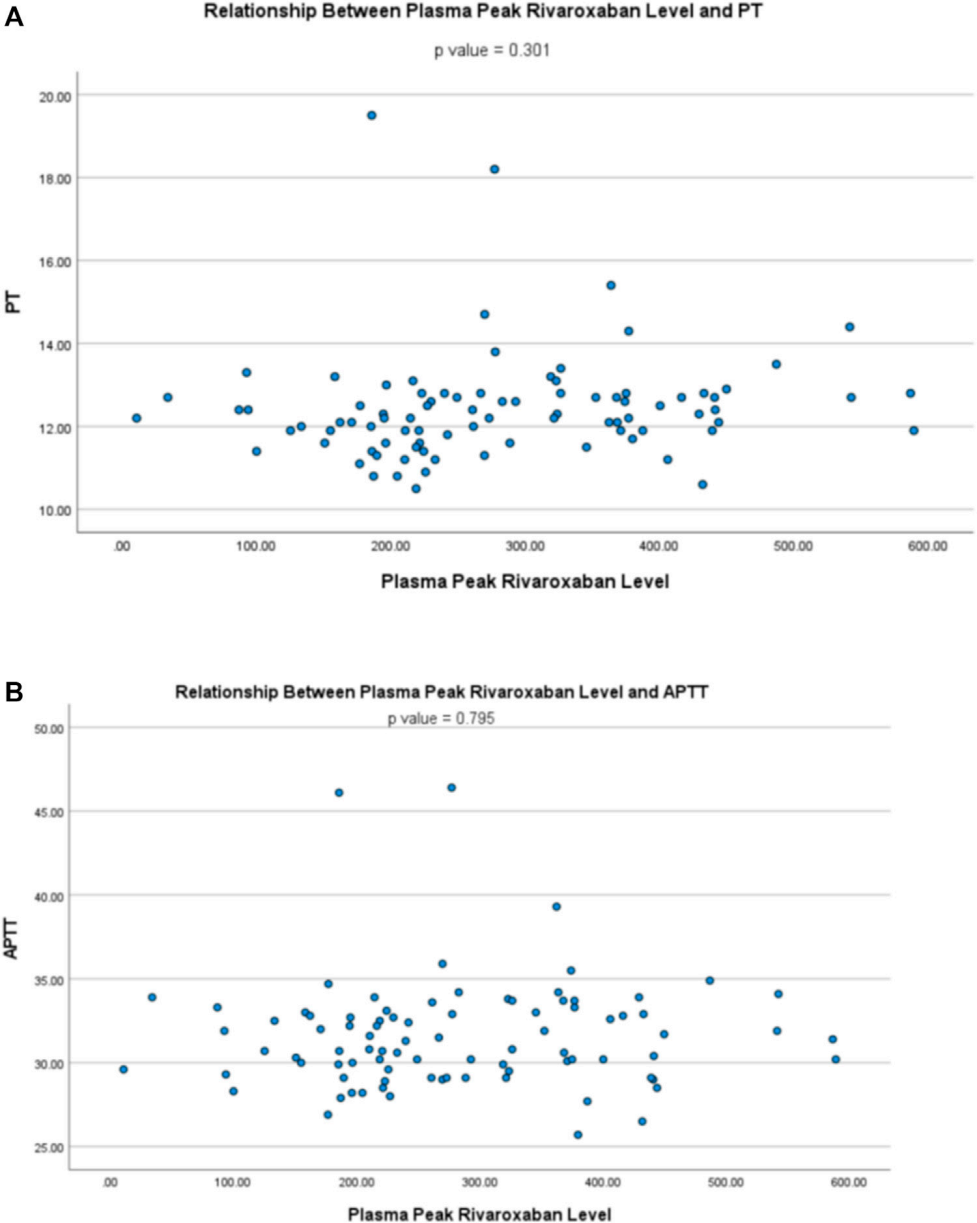
Relationship between plasma peak rivaroxaban level and **(A)** prothrombin time (PT) and **(B)** activated partial thromboplastin time (APTT).

The plasma trough rivaroxaban level showed a positive correlation with PT (r = 0.646, *p* < 0.001) and APTT (r = 0.437, *p* < 0.001) (Figures 5A,B), while the peak level showed no relationship with both PT (r = 0.111, *p* = 0.301) and APTT (r = 0.028, *p* = 0.795) (Figures 6A,B).

### Relationship Between Plasma Rivaroxaban Level and Clinically Significant Events

The median follow-up period was 36.1 months (range: 1–45 months). There was a total of 27 episodes of bleeding complications (annual rate of 7.7%) and five episodes of recurrent ischaemic/thromboembolic events (annual rate of 1.3%) during the entire follow-up period (Table 3). The median time from blood sample for plasma rivaroxaban level to occurrence of clinical events was 399.5 days (range: 24-950 days). The plasma trough rivaroxaban level showed a positive correlation with the rate of bleeding complications [odds ratio (OR): 1.018, 95% CI 1.004–1.033, *p* = 0.014] but not the rate of recurrent ischaemia/thromboembolic event (OR 0.976, 95% CI 0.935–1.018, *p* = 0.257) upon univariate regression analysis. Multi-variate regression analysis further demonstrated that plasma trough rivaroxaban level was positively associated with the rate of bleeding complications after adjustment for eGFR, age, dosage, history of DM, hypertension, ischaemic heart disease and ischaemic stroke (OR: 1.020, 95% CI 1.002–1.038, *p* = 0.028). Patients who developed bleeding complications also showed to have a significantly higher plasma trough rivaroxaban level than those who did not (59.9 ± 35.6 ng/ml vs. 41.1 ± 29.2 ng/ml, *p* = 0.011) (Table 4).

**TABLE 3:** Rate of clinically significant events in patients of different stages of CKD.

Outcome of rivaroxaban treatment		Rivaroxaban 15 mg daily	Rivaroxaban 20 mg daily	*p* value	Overall
**Total recurrent ischemic events (%)**	Stage 1 CKD	—	0 (0)	—	0 (0)
	Stage 2 CKD	1 (11.1)	2 (4.6)	—	3 (3.3)
	Stage 3 CKD	1 (6.7)	1 (7.1)	—	2 (2.2)
	Overall	2 (8.3)	3 (4.4)	0.603	5 (5.4)
**Stroke/Transient ischemic attack (%)**	Stage 1 CKD	—	0 (0)	—	0 (0)
	Stage 2 CKD	1 (11.1)	1 (2.3)	—	2 (2.2)
	Stage 3 CKD	1 (6.7)	1 (7.1)	—	2 (2.2)
	Overall	2 (8.3)	2 (2.9)	—	4 (4.3)
**Deep vein thrombosis (%)**	Stage 1 CKD		0 (0)	—	0 (0)
	Stage 2 CKD	0 (0)	1 (2.3)	—	1 (1.1)
	Stage 3 CKD	0 (0)	0 (0)	—	0 (0)
	Overall	0 (0)	1 (1.5)	—	1 (1.1)
**Bleeding complications (%)**	Stage 1 CKD	0 (0)	1 (10)	—	1 (10)
	Stage 2 CKD	3 (33.3)	15 (34.1)	—	18 (34.0)
	Stage 3 CKD	5 (33.3)	3 (21.5)	—	8 (27.6)
	Overall	8 (33.3)	19 (27.9)	0.482	27 (29.3)
**Major Bleeding n (%)**	Stage 1 CKD	0 (0)	0 (0)	—	0 (0)
	Stage 2 CKD	1 (11.1)	4 (9.1)	—	5 (9.4)
	Stage 3 CKD	1 (6.7)	1 (7.1)	—	2 (6.9)
**Minor Bleeding n (%)**	Stage 1 CKD	0 (0)	1 (10)	—	1 (10)
	Stage 2 CKD	2 (22.2)	11 (25)	—	13 (24.5)
	Stage 3 CKD	4 (26.7)	2 (14.3)	—	6 (20.7)

**TABLE 4:** Mean peak and trough plasma rivaroxaban level in patients with or without bleeding events and recurrent ischaemic/thromboembolic events.

Occurrence of events		Yes	No	*p* value
		(mean ± SD)		
Bleeding events	Peak rivaroxaban level	278.2 ± 122.8	286.9 ± 123.6	1.00
	Trough rivaroxaban level	59.9 ± 35.6	41.1 ± 29.2	0.011
Recurrent ischaemic/thrombotic events	Peak rivaroxaban level	230.0 ± 120.0	287.6 ± 123.2	0.336
	Trough rivaroxaban level	30.3 ± 14.8	47.6 ± 32.7	0.348

The peak plasma rivaroxaban level did not show any relationship with the rate of recurrent ischaemic/thromboembolic events and bleeding complications (*p* > 0.05 for both). Nevertheless, patients with recurrent ischaemic/thromboembolic events showed lower peak (230.0 ± 120.0 ng/ml vs. 287.6 ± 123.2 ng/ml, *p* = 0.336) and trough plasma rivaroxaban levels (30.3 ± 14.8 ng/ml vs. 47.6 ± 32.7 ng/ml, *p* = 0.348) compared with those who did not. The plasma peak rivaroxaban levels were similar in patients with or without bleeding complications (278.2 ± 122.8 ng/ml vs. 286.9 ± 123.6 ng/ml respectively, *p* = 1.00) (Table 4).

## Discussion

Rivaroxaban is licensed in patients with non-valvular AF whose CrCl are >15 ml/min (Jain and Reilly, 2019), and therefore a substantial number of patients receiving rivaroxaban worldwide have early stages of CKD. The use of rivaroxaban in CKD patients, despite dosage reduction, can be challenging as renal clearance accounts for approximately 36% of drug elimination. Here we observed an inverse relationship between the plasma trough level and eGFR in patients receiving rivaroxaban 20 mg daily. In the real clinical setting, patients with lower eGFR are often prescribed rivaroxaban 15 mg daily instead of 20 mg daily. In this cohort, 54.2% of patients who received rivaroxaban 15 mg daily had eGFR more than 50 ml/min but their eGFRs were significantly lower than the 20 mg group. Although our data showed 22 out of 68 patients (32.4%) who receiving 20 mg daily should take a reduced dose of 15 mg daily instead by calculating eGFR by Cockcroft-Gault formula, our present data suggests that despite receiving a lower dosage, patients who take 15 mg daily show statistically higher trough level than those taking 20 mg daily. The mean peak level was also numerically higher in the 15 mg group compared with the 20 mg group. These findings highlight the impact of renal impairment on drug accumulation and exposure of rivaroxaban. In this study, the plasma trough level of patients receiving rivaroxaban 20 mg daily was inversely correlated with eGFR and the levels in patients with stage 3 CKD were significantly higher than those with earlier stages of CKD. Such association was less obvious for peak drugs levels and those receiving rivaroxaban 15 mg daily. Compared with peak drug level, trough drug levels are more affected by renal impairment because of drug accumulation (Turpie et al., 2017). Rivaroxaban prescribed as fixed dose with adjustment of dosage according to renal function and routine measurement of plasma rivaroxaban level is not a standard practice currently (Douxfils et al., 2018), but such practice may be more worthwhile in certain patient subgroups such as those with CKD. In a Japanese study, the peak anti-Xa level of patients with severe renal impairment (CrCl 15-29 ml/min) was significantly higher than that of moderate renal impairment (CrCl 30-49 ml/min) (Tobe et al., 2020). Another Caucasian study reported that the plasma rivaroxaban level was positively correlated with worsening eGFR (Seiffge et al., 2018). Our results suggest that therapeutic drug monitoring of rivaroxaban may help optimize dosage in CKD patients. In this context, the trough level appears to show better correlation with kidney function than the peak level. Another advantage of measuring trough level is patient convenience because this can be measured along with other fasting blood samples.

Our data also demonstrated that plasma trough rivaroxaban level correlated with PT and APTT. This is of clinical relevance because measurement of plasma rivaroxaban concentration is expensive and often not routinely available in many clinical laboratories (Sarode, 2019). Therefore, our observations suggest that common clotting parameters such as PT and APTT can be useful surrogates to alert clinicians of patients with excessive rivaroxaban exposure and streamline the application of rivaroxaban level monitoring. Recommendations had been made on the utility of PT and APTT in excluding the presence of supra-therapeutic level of rivaroxaban (Conway et al., 2017; Douxfils et al., 2018). However, one should appreciate that the degree of PT prolongation depended on the reagent used (Conway et al., 2017; Sarode, 2019), and thus PT should not be used to substitute direct quantitation of plasma rivaroxaban level. Similarly, the sensitivity and response of APTT at different plasma concentration of rivaroxaban are reagent-dependent and hence APTT should not replace therapeutic drug monitoring of rivaroxaban (Conway et al., 2017). The correlation between trough but not peak levels of rivaroxaban, PT and APPT may be explained by the relatively small sample size in this study. Other possible explanations for such observation includes the curvilinear relationship between rivaroxaban levels and PT and APTT at a high plasma rivaroxaban level as demonstrated by other investigators (Dale et al., 2014; Siegal and Konkle, 2014). Therefore, the plasma peak rivaroxaban level did not show significant correlation with PT and APTT from our study.

Our data suggests that there may be an association between plasma trough rivaroxaban level and the rate of bleeding complication, and that patients who developed bleeding complications in this cohort appeared to have higher trough rivaroxaban levels than those who did not. Indeed, risk of haemorrhage due to accumulation of rivaroxaban in plasma is a valid concern in Chinese CKD patients, as various studies have reported a higher bleeding risk in Chinese compared with Caucasians (Shen et al., 2007; Tsai et al., 2013; Wong et al., 2014). The risk of bleeding may also be further exacerbated by long-standing hypertension and platelet dysfunction which are often present in CKD patients. The relationship between rivaroxaban levels and bleeding is intriguing. Several Caucasian studies demonstrated the relationship between plasma DOAC level and risk of haemorrhagic complications. Bernier et al. showed that risk of bleeding complications was associated with higher than the expected range of rivaroxaban level obtained from clinical trials (Bernier et al., 2020). Sennesael, A.L. et al. had demonstrated that patients with major bleeding had a high DOAC level at the time of presentation (Sennesael et al., 2018). Testa et al. showed elevated plasma peak DOAC level was associated with increased bleeding risk (Testa et al., 2019). Here we did not observe any relationship between plasma rivaroxaban level and the occurrence of recurrent ischaemic/thromboembolic events, although these patients tend to show numerically lower drug levels than those who did not. This is not surprising as many other vascular risk factors such as diabetes, hypertension, dyslipidaemia and aberrant vascular anatomy also plays important roles in the pathogenesis of recurrent ischaemic/thromboembolic events.

Limitations of this study include the relatively small sample size and all patients are of Chinese ethnic origin. Furthermore, the wide ranges of time between measurement of blood rivaroxaban, the time of initiation of rivaroxaban and the occurrence of clinically significantly events render a definite conclusion on blood rivaroxaban levels and clinical events difficult. Moreover, patients with advanced CKD were excluded from this study and the overall event rates were low. Another shortcoming is that the plasma rivaroxaban levels were measured as part of the routine blood monitoring during stable disease but not exactly at the time of clinically significant events. Nevertheless, our study provides important clinical data regarding the drug exposure of rivaroxaban in early CKD patients who have increased susceptibility to drug accumulation and thus its side effects. Larger prospective studies that evaluate the full pharmacokinetics profile of rivaroxaban in CKD patients and its relationships with treatment efficacy and adverse events will help define dosages in patients with renal insufficiency.

## Data Availability

The original contributions presented in the study are included in the article/Supplementary Material, further inquiries can be directed to the corresponding author.
